# Bioreactance Is Not Interchangeable with Thermodilution for Measuring Cardiac Output during Adult Liver Transplantation

**DOI:** 10.1371/journal.pone.0127981

**Published:** 2015-05-27

**Authors:** Sangbin Han, Jong Hwan Lee, Gaabsoo Kim, Justin Sangwook Ko, Soo Joo Choi, Ji Hae Kwon, Burn Young Heo, Mi Sook Gwak

**Affiliations:** Department of Anesthesiology and Pain Medicine, Samsung Medical Center, Sungkyunkwan University School of Medicine, Seoul, Korea; University of Toledo, UNITED STATES

## Abstract

**Background:**

Thermodilution technique using a pulmonary artery catheter is widely used for the assessment of cardiac output (CO) in patients undergoing liver transplantation. However, the unclearness of the risk-benefit ratio of this method has led to an interest in less invasive modalities. Thus, we evaluated whether noninvasive bioreactance CO monitoring is interchangeable with thermodilution technique.

**Methods:**

Nineteen recipients undergoing adult-to-adult living donor liver transplantation were enrolled in this prospective observational study. COs were recorded automatically by the two devices and compared simultaneously at 3-minute intervals. The Bland–Altman plot was used to evaluate the agreement between bioreactance and thermodilution. Clinically acceptable agreement was defined as a percentage error of limits of agreement <30%. The four quadrant plot was used to evaluate concordance between bioreactance and thermodilution. Clinically acceptable concordance was defined as a concordance rate >92%.

**Results:**

A total of 2640 datasets were collected. The mean CO difference between the two techniques was 0.9 l/min, and the 95% limits of agreement were -3.5 l/min and 5.4 l/min with a percentage error of 53.9%. The percentage errors in the dissection, anhepatic, and reperfusion phase were 50.6%, 56.1%, and 53.5%, respectively. The concordance rate between the two techniques was 54.8%.

**Conclusion:**

Bioreactance and thermodilution failed to show acceptable interchangeability in terms of both estimating CO and tracking CO changes in patients undergoing liver transplantation. Thus, the use of bioreactance as an alternative CO monitoring to thermodilution, in spite of its noninvasiveness, would be hard to recommend in these surgical patients.

## Introduction

Liver transplantation (LT) is accompanied by rapid intraoperative hemodynamic changes due to clamping/unclamping of the major abdominal vessels, sudden blood loss, rapid fluid infusion, postreperfusion syndrome, and embolic events [1]. These surgical events are further complicated by the intrinsic circulatory alteration of cirrhotic patients featuring low systemic vascular resistance (SVR), high cardiac output (CO), and cirrhotic cardiomyopathy [1]. Thus, intensive intraoperative assessment of preload, afterload, and cardiac function is essential; thermodilution via pulmonary artery catheter (PAC) has long been the clinical gold standard in estimating CO, SVR, pulmonary arterial pressures, and ventricular filling pressure [2]. However, insufficient evidence that pulmonary artery catheterization improves post-transplant clinical outcomes and its complications, such as arrhythmias [3], pulmonary artery injury, thrombosis, and infection have contributed to a trend in favor of less invasive monitoring. Although the PAC is still widely used clinically [4], some anesthesiologists rely on central venous pressure alone and others utilize transesophageal echocardiography (TEE), calibrated pulse pressure analysis (PiCCO and LiDCO), pulse contour analysis (FloTrac), or ultrasound Doppler monitoring (USCOM) as alternatives to thermodilution. Validation of these less invasive modalities is ongoing [1, 5–7].

As an easy-to-use noninvasive CO monitoring device, NICOM (Cheetah Medical Inc., Wilmington, Delaware) utilizes thoracic bioreactance to analyze the variations in voltage in each cardiac contraction in response to high-frequency current. Previous studies in various populations have reported mixed results regarding the performance of bioreactance for measuring CO [8–13]. However, no studies have evaluated this technique in liver transplant recipients. Thus, this study evaluated whether bioreactance can be used as an alternative CO monitoring technique to thermodilution during LT.

## Methods

### Subjects and study protocols

This prospective observational study was approved by the Institutional Review Board (SMC 2012-08-077) of our institution and was registered with cris.nih.go.kr (KCT0000596). Written informed consent was obtained from 20 recipients who underwent elective adult-to-adult living donor LT using the right hemiliver graft. There were no exclusion criteria.

The clock times of the bioreactance monitor and the thermodilution monitor were synchronized to the network time signal prior to surgery in order to obtain simultaneous reading from both devices. Each bioreactance electrode sticker (NICOM, Cheetah Medical Inc., Wilmington, Delaware) is comprised of one high-frequency (75 kHz) current generator and one voltage input amplifier (or voltage receiver). Before anesthetic induction, four electrode stickers were placed on the recipients: two upper electrode stickers in the bilateral midsubclavian regions and their paired lower electrode stickers at the bilateral 12th ribs on the posterior axillary line. All electrode stickers were secured by a transparent film dressing. After the initial calibration was automatically performed at the start of the system, internal calibration was manually performed at the end of the skin incision and hourly thereafter as well as when significant hemodynamic changes were anticipated (e.g. clamping of major vessels and significant position changes). Bioreactance-derived COs were automatically recorded in the build-in hard driver at 1-minute intervals. The electrode stickers were removed from the patients at the end of surgery.

A PAC (Swan-Ganz CCOmbo V, Edwards LifeSciences, LLC, Irvine, CA) was introduced through the right internal jugular vein in combination with a 9-Fr large-bore central venous catheter and threaded into the pulmonary artery (2 cm behind the point of pulmonary artery occlusion by the tip balloon). The position of the PAC tip was adjusted to a signal quality indicator of 1 throughout the surgery. Thermodilution-derived COs were automatically recorded into the hard driver via a data logger at 1-minute intervals, together with other indices including central venous pressure, end-diastolic right ventricular volume, mixed venous oxygen saturation (SvO2), stroke volume, heart rate, right ventricular ejection fraction, and body core temperature. CO measured by STAT-mode was used for the analysis because STAT-mode uses the same time frame to the bioreactance and provide COs that are time averaged over the preceding 1 minute, whereas CCO-mode is averaged over a 3-minute time frame. In addition, a 1-minute time frame was considered more clinically relevant to reflect frequent, rapid, and wide hemodynamic changes during LT, as discussed previously [1]. The PAC was removed from the patients at the end of surgery.

The agreement in COs between the two techniques was evaluated with datasets obtained at 3-minute intervals. COs obtained after the end of the skin incision were used for analysis because the skin incision started shortly after obtaining the first thermodilution-derived CO value, and bioreactance-derived CO could not be obtained during the skin incision due to persistent electrocauterization-induced signal interference for more than 40 seconds in a given minute [14]. STAT-mode did not measure COs immediately after the graft reperfusion (around 5 minutes in general) due to the thermal disturbances and consequent lack of a baseline pulmonary arterial temperature, and thus, analysis was not performed during this period.

### Anesthetic managements

Anesthetic management was performed according to our institutional LT protocol. Anesthesia was induced with thiopental sodium (5mg/kg) and maintained with isoflurane titrated to a bispectral index of 40–60. Neuromuscular blockade was achieved using a continuous infusion of vecuronium at 0.8–1.0 μg/kg/min. Mechanical ventilation was delivered at a tidal volume of 8–10 ml/kg using a mixture of medical air and oxygen at a fresh gas flow rate of 2 l/min. The respiratory rate was adjusted as needed to maintain normocapnea. Direct blood pressure monitoring was performed via the right radial artery, right femoral artery, right femoral vein, and right internal jugular vein. Infusions of fluids and rescue drugs, such as dopamine and norepinephrine, aimed to maintain mean arterial pressure ≥ 70 mmHg and systemic vascular resistance > 600 dyn s/cm^5^. Normothermia was maintained using a whole-body convective warming blanket, heat-and-moisture exchanger, room temperature thermostatically set at 24^°^C, vinyl arm wraps, and a rapid fluid warmer.

### Surgical procedures

All grafts consisted of liver segments 5 to 8 excluding the middle hepatic vein trunk. After procurement, the graft was perfused through the portal vein and hepatic artery with histidine tryptophan ketoglutarate solution until the perfusate was clear. Graft implantation was performed using the piggyback technique. After portal vein anastomosis, the graft was reperfused by consecutively unclamping the hepatic vein and portal vein. Subsequently, hepatic artery anastomosis was performed, followed by biliary anastomosis.

### Statistical analyses

The agreement between the two techniques was analyzed using the Bland–Altman plot, which was a difference (thermodilution minus bioreactance) versus average (thermodilution/2 + bioreactance/2) plot, with multiple observations per individual [15]. The difference in CO between the two techniques was summarized as the mean ± 1.96 standard deviation (SD), which are so-called 95% limits of agreement, and the percentage error of the limits of agreement [15]. The percentage error was calculated as 1.96 SD divided by the mean value of the average and was considered clinically acceptable if it was < 30%, as described previously [16]. A four-quadrant plot was used to assess the concordance of directional changes between consecutive points measured from both devices. To improve the validity of the statistical analysis, a 15% exclusion zone for small changes in CO was applied [17]. The concordance rate was a measure of the proportion of data points where bioreactance-derived CO and thermodilution-derived CO changed in the same direction and was considered clinically acceptable if it was > 92% [17]. The associations between the distance in CO between the two techniques and the indices which were measured via PAC and recorded simultaneously with thermodilution-derived CO were analyzed using the Pearson correlation. Continuous variables were described as the mean ± SD or median (25th percentile-75 percentile, minimum-maximum), as appropriate. Categorical variables were presented as number (%). All reported *P* values were two-sided and *P* < 0.05 was considered statistically significant. SPSS 20.0 (SPSS Inc., Chicago, IL, USA) was used for the statistical analyses.

## Results

There was one patient dropout due to early inadvertent bioreactance electrode disconnection. Data from 19 recipients with 2640 datasets was ultimately included. The indications for LT were hepatocellular carcinoma with viral cirrhosis (n = 15), cirrhosis secondary to viral etiology (2), and alcoholic cirrhosis (2). The clinical data of the 19 recipients are shown in Table 1. The median Model for End-stage Liver Disease (MELD) score was 13. All recipients showed sinus cardiac rhythm and none of them had a preoperative peak in right ventricular pressure of ≥ 40 mmHg. The mean thermodilution-derived CO was 8.7 ± 2.5 l/min and the mean bioreactance-derived CO was 7.8 ± 2.4 l/min.

**Table 1:** Preoperative and intraoperative clinical data of liver transplant recipients.

Variables	Descriptive statistics
Age (years)	55 (51–60, 38–68)
Female/male (number)	4/15
Body mass index (kg/m^2^)	24.6 (21.7–26.1, 17.5–35.3)
Parenchymal lung disease (number)	0
Pulmonary function tests	
Forced expiratory volume in 1 second (FEV1, %)	86 (81–101, 57–116)
FEV1 to forced vital capacity ratio (%)	77 (72–83, 53–87)
Preoperative echocardiography at rest	
Arrhythmia (number)	0
Left ventricle ejection fraction (%)	68 (62–69, 58–76)
Peak right ventricular pressure (mmHg)*	25 (23–30, 19–38)
Valvular disease (mild/moderate/severe)	1/1/0
Wall motion abnormality (number)	1/1/0
Baseline thermodilution-derived variables (at skin incision)	
Cardiac output (l)	5.7 (4.9–6.9, 3.8–9.7)
Systemic vascular resistance (dyn sec/cm^5^)	902 (732–1077, 461–1337)
Heart rate (/min)	68 (64–71, 60–95)
Mean arterial pressure (mmHg)	72 (63–78, 60–90)
Mean pulmonary arterial pressure (mmHg)	13 (11–16, 6–27)
Right ventricular end-diastolic volume (ml)	258 (230–313, 154–350)
Right ventricular ejection fraction (%)	32 (29–38, 20–48)
SvO^2^ (%)†	88.6 (84.0–89.6, 81.6–93.0)
Model for End-stage liver Disease score	13 (8–16, 7–32)
Graft-to-recipient weight ratio (%)	1.09 (0.94–1.33, 0.80–1.70)
Anhepatic time (minutes)	138 (115–153, 80–250)
Operative time (minutes)	580 (538–620, 411–798)
Crystalloid (ml/hr)‡	709 (650–823, 547–1198)
Red blood cells salvaged by the cell saver (ml)	689 (485–1399, 227–7094)
Autotransfusion of salvaged red blood cells (ml)	510 (0–1221, 0–7094)
Red blood cell transfusion (units)	0 (0–3, 0–7)
Analyzed datasets (dissection/anhepatic/reperfusion phases)	794/638/1208

Data are described as median (IQR, range) or number.

*Based on the tricuspid regurgitant jet.

†Measured via blood sampling.

‡Including Hartman's solution, plasma solution, dextrose solution, normal saline, and half saline.

**Fig 1:**
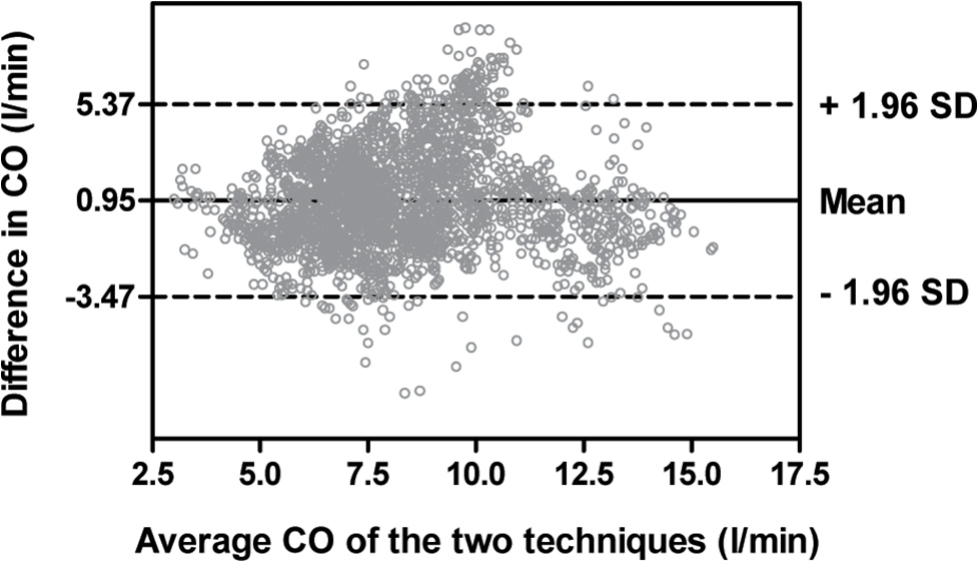
The Bland-Altman plot shows wide 95% limits of agreement between bioreactance and thermodilution in cardiac output (CO).

**Fig 2:**
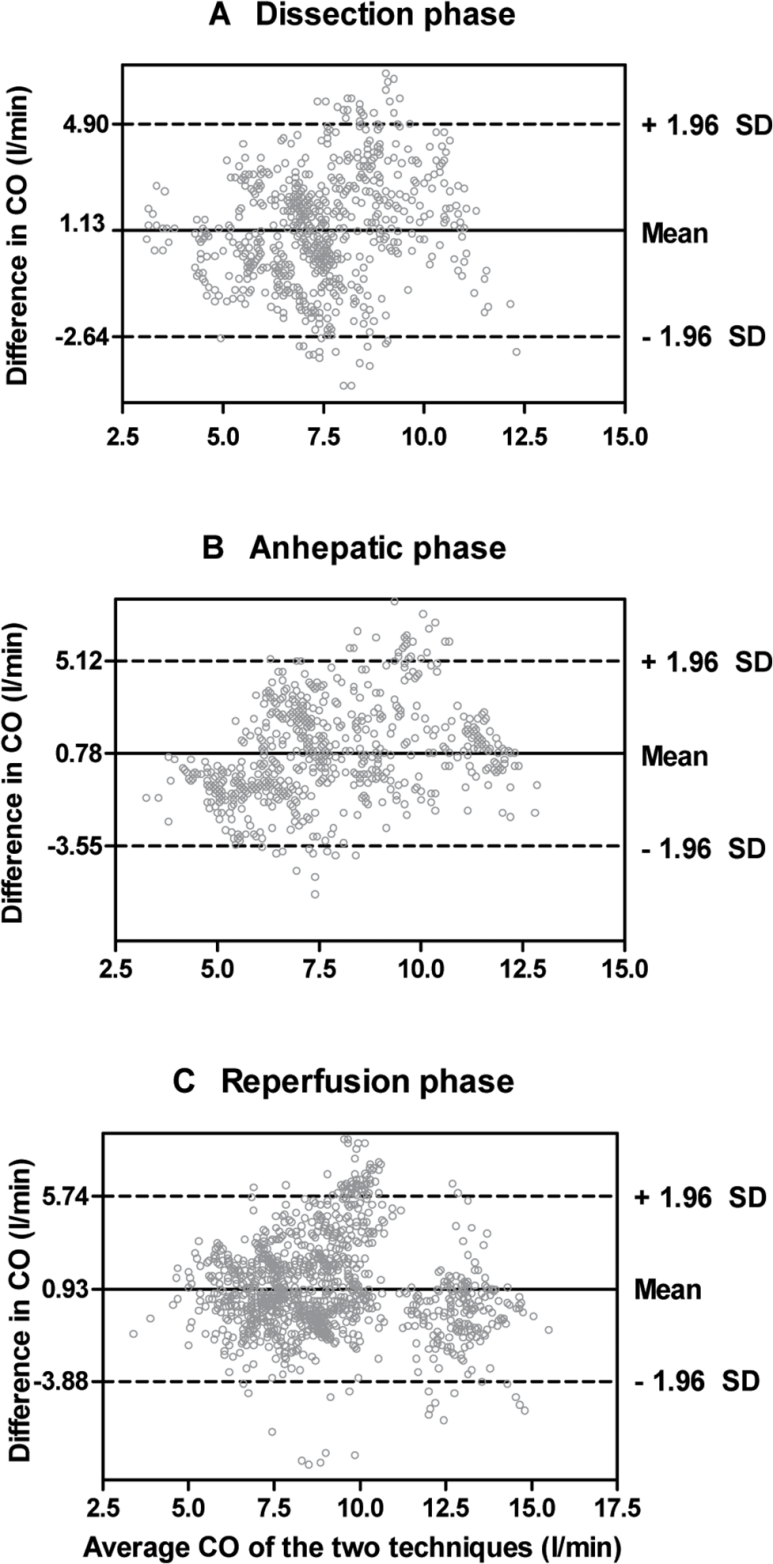
The Bland-Altman plot shows wide 95% limits of agreement between bioreactance and thermodilution in cardiac output (CO) during the dissection (A), anhepatic (B), and reperfusion phase (C).

**Fig 3:**
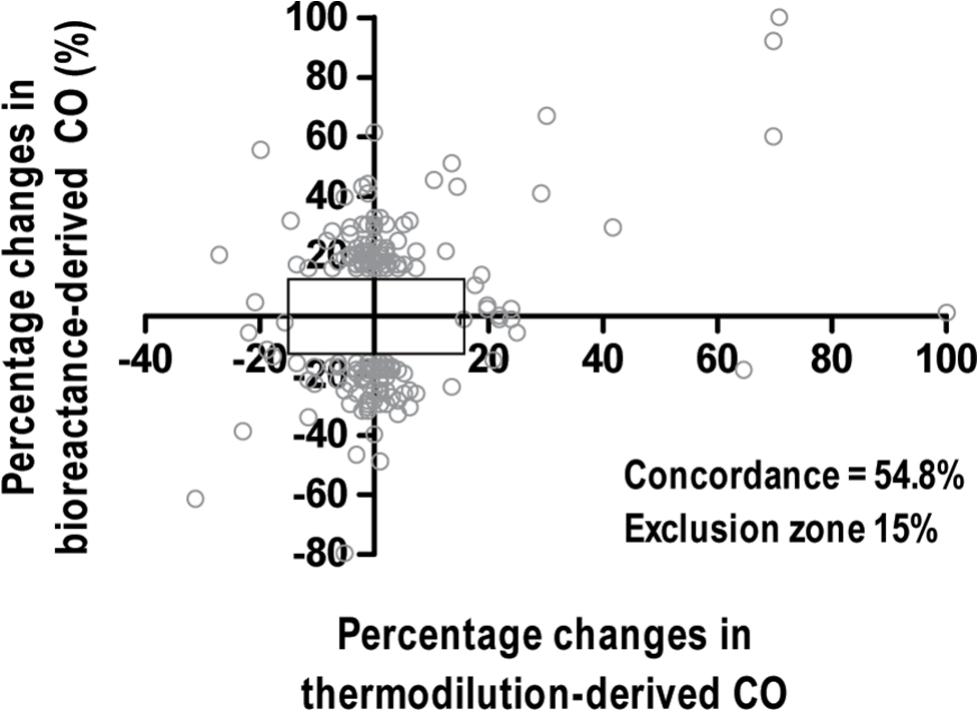
Four-quadrant concordance analysis between the changes in cardiac output (CO) measured by bioreactance and thermodilution. The zero-centered square corresponds to the 15% exclusion zone.

The mean difference between the two techniques was 0.95 l/min and 95% limits of agreement were -3.47 and 5.37 l/min (Fig1). The percentage error of the limits of agreement was 53.8%. The proportion of datasets with < 1.0 l/min difference was 39.0%. A total of 2640 datasets were divided into three subgroups according to operative phase (794 datasets for the dissection phase, 638 datasets for the anhepatic phase, and 1208 datasets for the reperfusion phase). As shown in Fig 2, the agreement between the two techniques was not satisfactory throughout the operative phases. The percentage error in the dissection, anhepatic, and reperfusion phase was 50.6%, 56.1%, and 53.5%, respectively. As shown in Fig 3, a total of 199 datasets were included for the concordance analysis after applying the 15% exclusion zone. The concordance rate of CO changes between the two techniques was 54.8%.

The distance in CO between the two techniques was positively correlated with stroke volume (Pearson coefficient [γ] = 0.55, *P* < 0.001), right ventricular ejection fraction (γ = 0.22, *P* < 0.001), end-diastolic right ventricular volume (γ = 0.35, *P* < 0.001), and SvO_2_ (γ = 0.25, *P* < 0.001). That was negatively correlated with SVR (γ = -0.40, *P* <0.001) and body core temperature (γ = -0.11, *P* < 0.001).

## Discussion

Bioreactance is a novel CO measuring technique with advantage of noninvasiveness. NICOM is the only commercially available bioreactance CO monitoring system and has been validated in various clinical settings. This study is the first to evaluate the intraoperative performance of NICOM in the clinical LT setting. Intrapulmonary thermodilution via PAC is the established standard and was considered as the reference technique [2]. Recipients analyzed in the study had mild to moderate degree end-stage liver disease and none of them had cardiac arrhythmia, impaired contractility, regional wall motion abnormality, severe degree valve disease, or portopulmonary hypertension as well as parenchymal lung disease. A completely automatic and continuous data-collection was used for both thermodilution and bioreactance. Our observation showed unacceptable agreement between bioreactance and thermodilution for assessing CO, demonstrated by wide 95% limits of agreement. We consistently observed percentage errors > 50% throughout the surgery (during the dissection, anhepatic, and reperfusion phases). The concordance rate was also far lower than the recommended lower limit of 92%. The distance between the two techniques increased in relation to hyperdynamic circulatory conditions, which are common in liver transplant recipients, such as greater stroke volume, right ventricular ejection fraction, end-diastolic right ventricular volume, and SvO_2_ and lower systemic vascular resistance. The agreement level was also affected by body temperature, which tends to fluctuate widely during LT. Overall, bioreactance cannot be considered interchangeable with thermodilution via PAC to assess CO during LT.

Bioreactance measures CO based on the analysis of the difference in amplitude between the injected signal that traverses the thoracic cavity and the output signal received from the thorax, which depends on the amount of fluids in the thorax, and phase shift, which is the instantaneous change in the direction of impedance and is generated only by pulsatile flow. Because the majority of pulsatile flows originates from the aorta, bioreactance can efficiently identify the changes in the amount of water in the aorta. In short, stroke volume is calculated using the following formula: C VET dФ/d*t*_max_, where C is a constant of proportionality determined by patient age, gender and body size, VET is ventricular ejection time determined from the electrocardiographic signals, and dФ/d*t*_max_ is the peak rate of change of the relative phase shift (Ф) determined by a highly sensitive phase detector. This signal processing is developed to overcome the disadvantages of the traditional bioimpedance which relies only on the amplitude without consideration of phase, and accordingly, is more affected by patient body size, movement, electrodes position, and physical factors that influence electrical conductivity between the electrodes and skin (e.g. temperature and humidity) [8, 14].

Indeed, bioreactance has shown excellent performance for measuring CO and tracking CO changes during and after cardiac surgery, in the medical intensive care unit, and even in patients with pulmonary hypertension [8, 12, 13]. However, this technique has failed to obtain acceptable accuracy and precision for measuring CO during major abdominal surgeries and in patients with septic shock with the percentage error of limits of agreement ranging from 50.7%-82.0% compared to those of thermodilution [9–11, 18]. These studies and the current study might have similar mechanisms underlying unacceptable agreement between bioreactance and thermodilution because LT is a major open abdominal surgery, and end-stage liver disease patients have hyperdynamic circulation analogous to that of septic shock. First, high intravenous volume administration is necessary during LT due to fluid loss via the opened surgical field and fluid shift into the extravascular space [10]. More rapid administration of relatively large amount of fluids occurs frequent due to hemostatic imbalance, portosystemic collaterals, clamping of major vessels, and vascular anastomoses. Furthermore, end-stage liver disease patients suffer from reduced vascular osmotic pressure, disrupted endovascular barrier, ascites, and increased plasma volume [19]. Taken together, these factors can result in fluid accumulation within the thoracic tissues, and the change in the underlying level of thoracic fluid induces variation in thoracic impedance and might limit the performance of the device for measuring CO [20]. Second, the bioreactance signal was still affected by patient body temperatures despite the improvement of the processing algorithm. Transplant recipients face challenging thermal environments and significant intraoperative body core/skin temperature changes during surgery [21, 22]. It can be assumed that the changes in underlying body temperatures and the difference between skin and core temperature affected the results of this study. Third, chest retraction and frequent patient position change are mandatory for surgical procedures and these maneuvers affect baseline lung volume and could alter the bioreactance reading [9]. Fourth, thoracic impedance is negatively correlated with the iron content in the thorax; thus, blood loss and red blood cell transfusion would affect thoracic impedance and relative phase shifts [9]. Fifth, hyperdynamic circulation in liver transplant recipients is complicated, displaying high cardiac output, increased compliance of the arteries including the aorta, arteriovenous communications, redundant collateral system, and altered CO distribution into organs [1, 19, 23]. All these findings necessitate derivation and validation of the specific algorithm for liver transplant recipients (e.g. a proportionality constant different from that of the general population), which has not yet been developed.

The study has limitations. First, the sample size was small and further validation of the present study in large transplant populations is warranted. Second, the intermittent bolus thermodilution was not used as the reference parameter for CO. We defined the time interval between adherent datasets as 3 minutes in comparison to previous studies adopting only predetermined time points at which greater hemodynamic changes were anticipated. This was because intraoperative hemodynamic changes can occur any time during LT and are often highly unpredictable. Accordingly, intermittent thermodilution, which requires three to five consecutive injections of 10 ml cold saline to acquire one CO value, was impractical due to concerns of portal hypertension, dilution coagulopathy, hypothermia, and bleeding. Moreover, continuous thermodilution and intermittent thermodilution have shown good agreement during LT [24, 25]. Third, we could not determine the mechanistic background underlying the unacceptable performance of bioreactance although we suggested that hyperdynamic circulation and low body core temperature contributed to this difference. It was not possible to evaluate the association between recipient physical characteristics and the performance of bioreactance due to the small sample size. Fourth, we did not continue CO monitoring in the intensive care unit after the surgery. Our transplant team routinely removed the PAC at the end of surgery due to its potential risks and relatively stable postoperative hemodynamic condition. Fifth, other indices measured by bioreactance were not evaluated in the current study. For example, thoracic fluid content and its percentage change have shown promising results in hemodynamic managements of patients undergoing hemodialysis and in pediatric patients undergoing cardiac surgeries who are at greater risk for volume overload or depletion [26, 27]. The use of these variables as either alternatives or adjuvants of thermodilution-derived variables requires further study. Also, inadequate performances of bioreactance for measuring CO in adult liver transplant recipients may not be transferable to pediatric liver transplantation because pediatric recipients are thought to reflect less hyperdynamic circulatory status. Given the impossibility of placing pulmonary artery catheter and purported promising performance of bioreactance in pediatric patients [28, 29], pediatric transplant recipients would be the population of particular interest.

In conclusion, bioreactance did not demonstrate adequate performance to replace invasive thermodilution via PAC in terms of both estimating CO and tracking CO changes in adult patients undergoing liver transplantation. Thus, the use of bioreactance as an alternative CO monitoring to thermodilution, in spite of its noninvasiveness, may not be recommended in this group of patients.

## References

[1] De Wolf AM. 6/2/06 Perioperative assessment of the cardiovascular system in ESLD and transplantation. Int Anesthesiol Clin. 2006;44(4):59–78. Epub 2006/10/13. 10.1097/01.aia.0000210818.85287.de . 17033479

[2] De Wolf AM. Pulmonary artery catheter: rest in peace? Not just quite yet. Liver Transpl. 2008;14(7):917–8. Epub 2008/06/27. 10.1002/lt.21543 . 18581507

[3] Gwak MS, Kim JA, Kim GS, Choi SJ, Ahn H, Lee JJ, et al Incidence of severe ventricular arrhythmias during pulmonary artery catheterization in liver allograft recipients. Liver Transpl. 2007;13(10):1451–4. 10.1002/lt.21300 . 17902132

[4] Schumann R, Mandell MS, Mercaldo N, Michaels D, Robertson A, Banerjee A, et al Anesthesia for liver transplantation in United States academic centers: intraoperative practice. J Clin Anesth. 2013;25(7):542–50. Epub 2013/09/03. 10.1016/j.jclinane.2013.04.017 . 23994704

[5] Wong LS, Yong BH, Young KK, Lau LS, Cheng KL, Man JS, et al Comparison of the USCOM ultrasound cardiac output monitor with pulmonary artery catheter thermodilution in patients undergoing liver transplantation. Liver Transpl. 2008;14(7):1038–43. Epub 2008/06/27. 10.1002/lt.21483 . 18581504

[6] Krejci V, Vannucci A, Abbas A, Chapman W, Kangrga IM. Comparison of calibrated and uncalibrated arterial pressure-based cardiac output monitors during orthotopic liver transplantation. Liver Transpl. 2010;16(6):773–82. Epub 2010/06/03. 10.1002/lt.22056 . 20517912

[7] Feltracco P, Biancofiore G, Ori C, Saner FH, Della Rocca G. Limits and pitfalls of haemodynamic monitoring systems in liver transplantation surgery. Minerva anestesiologica. 2012;78(12):1372–84. Epub 2012/08/04. . 22858882

[8] Keren H, Burkhoff D, Squara P. Evaluation of a noninvasive continuous cardiac output monitoring system based on thoracic bioreactance. American journal of physiology Heart and circulatory physiology. 2007;293(1):H583–9. Epub 2007/03/27. 10.1152/ajpheart.00195.2007 . 17384132

[9] Kupersztych-Hagege E, Teboul JL, Artigas A, Talbot A, Sabatier C, Richard C, et al Bioreactance is not reliable for estimating cardiac output and the effects of passive leg raising in critically ill patients. Br J Anaesth. 2013;111(6):961–6. Epub 2013/08/30. 10.1093/bja/aet282 . 23985531

[10] Kober D, Trepte C, Petzoldt M, Nitzschke R, Herich L, Reuter DA, et al Cardiac index assessment using bioreactance in patients undergoing cytoreductive surgery in ovarian carcinoma. Journal of clinical monitoring and computing. 2013;27(6):621–7. Epub 2013/05/22. 10.1007/s10877-013-9478-x . 23689837

[11] Conway DH, Hussain OA, Gall I. A comparison of noninvasive bioreactance with oesophageal Doppler estimation of stroke volume during open abdominal surgery: an observational study. European journal of anaesthesiology. 2013;30(8):501–8. Epub 2013/04/04. 10.1097/EJA.0b013e3283603250 . 23549128

[12] Rich JD, Archer SL, Rich S. Noninvasive cardiac output measurements in patients with pulmonary hypertension. The European respiratory journal. 2013;42(1):125–33. Epub 2012/10/27. 10.1183/09031936.00102212 . 23100501

[13] Cheung H, Dong Q, Dong R, Yu B. Correlation of cardiac output measured by non-invasive continuous cardiac output monitoring (NICOM) and thermodilution in patients undergoing off-pump coronary artery bypass surgery. Journal of anesthesia. 2014. Epub 2014/11/09. 10.1007/s00540-014-1938-z . 25381090 PMC4488496

[14] Marik PE. Noninvasive Cardiac Output Monitors: A State-of the-Art Review. J Cardiothorac Vasc Anesth. 2012. Epub 2012/05/23. 10.1053/j.jvca.2012.03.022 . 22609340

[15] Bland JM, Altman DG. Statistical methods for assessing agreement between two methods of clinical measurement. Lancet. 1986;1(8476):307–10. Epub 1986/02/08. . 2868172

[16] Critchley LA, Critchley JA. A meta-analysis of studies using bias and precision statistics to compare cardiac output measurement techniques. Journal of clinical monitoring and computing. 1999;15(2):85–91. Epub 2003/02/13. . 12578081 10.1023/a:1009982611386

[17] Critchley LA, Lee A, Ho AM. A critical review of the ability of continuous cardiac output monitors to measure trends in cardiac output. Anesth Analg. 2010;111(5):1180–92. 10.1213/ANE.0b013e3181f08a5b . 20736431

[18] Critchley LA, Peng ZY, Fok BS, James AE. The effect of peripheral resistance on impedance cardiography measurements in the anesthetized dog. Anesth Analg. 2005;100(6):1708–12. Epub 2005/05/28. 10.1213/01.ane.0000150602.40554.eb . 15920200

[19] Moller S, Henriksen JH. Cardiopulmonary complications in chronic liver disease. World J Gastroenterol. 2006;12(4):526–38. Epub 2006/02/21. ; PubMed Central PMCID: PMCPmc4066083. 16489664 10.3748/wjg.v12.i4.526 PMC4066083

[20] Peng ZY, Critchley LA, Fok BS. An investigation to show the effect of lung fluid on impedance cardiac output in the anaesthetized dog. Br J Anaesth. 2005;95(4):458–64. Epub 2005/07/30. 10.1093/bja/aei206 . 16051651

[21] Han S, Gwak MS, Choi SJ, Kim MH, Ko JS, Kim GS, et al Effect of active airway warming on body core temperature during adult liver transplantation. Transplant Proc. 2013;45(1):251–4. Epub 2013/02/05. 10.1016/j.transproceed.2012.05.088 . 23375310

[22] Han S, Gwak MS, Choi SJ, Ko JS, Kim GS, Son HJ, et al Risk factors for inadvertent hypothermia during adult living-donor liver transplantation. Transplant Proc. 2014;46(3):705–8. 10.1016/j.transproceed.2013.11.091 . 24767329

[23] Newby DE, Hayes PC. Hyperdynamic circulation in liver cirrhosis: not peripheral vasodilatation but 'splanchnic steal'. QJM: monthly journal of the Association of Physicians. 2002;95(12):827–30. Epub 2002/11/28. . 12454326 10.1093/qjmed/95.12.827

[24] Greim CA, Roewer N, Thiel H, Laux G, Schulte am Esch J. Continuous cardiac output monitoring during adult liver transplantation: thermal filament technique versus bolus thermodilution. Anesth Analg. 1997;85(3):483–8. Epub 1997/09/20. . 9296398 10.1097/00000539-199709000-00003

[25] Bottiger BW, Sinner B, Motsch J, Bach A, Bauer H, Martin E. Continuous versus intermittent thermodilution cardiac output measurement during orthotopic liver transplantation. Anaesthesia. 1997;52(3):207–14. Epub 1997/03/01. . 9124659 10.1111/j.1365-2044.1997.079-az0077.x

[26] Kossari N, Hufnagel G, Squara P. Bioreactance: a new tool for cardiac output and thoracic fluid content monitoring during hemodialysis. Hemodialysis international International Symposium on Home Hemodialysis. 2009;13(4):512–7. Epub 2009/09/18. 10.1111/j.1542-4758.2009.00386.x . 19758300

[27] Kang WS, Lee JH, Shin HJ, Kim SH, Kim TY, Seo DM, et al Noninvasive cardiac output monitoring in paediatric cardiac surgery: correlation between change in thoracic fluid content and change in patient body weight. J Int Med Res. 2012;40(6):2295–304. Epub 2013/01/17. PubMed 23321186. 23321186 10.1177/030006051204000627

[28] Weisz DE, Jain A, McNamara PJ, A EL-K. Non-invasive cardiac output monitoring in neonates using bioreactance: a comparison with echocardiography. Neonatology. 2012;102(1):61–7. Epub 2012/04/18. 10.1159/000337295 . 22508150

[29] Vergnaud E, Vidal C, Montmayeur Verchere J, Taright H, Meyer PG, Carli PA, et al. Noninvasive cardiac output measurement using bioreactance in postoperative pediatric patients. Paediatric anaesthesia. 2014. Epub 2014/05/13. 10.1111/pan.12412 . 24814690

